# Implementation of genomics research in Africa: challenges and recommendations

**DOI:** 10.1080/16549716.2017.1419033

**Published:** 2018-01-16

**Authors:** Sally N. Adebamowo, Veronica Francis, Ernest Tambo, Seybou H. Diallo, Guida Landouré, Victoria Nembaware, Eileen Dareng, Babu Muhamed, Michael Odutola, Teniola Akeredolu, Barbara Nerima, Petronilla J. Ozumba, Slee Mbhele, Anita Ghanash, Ablo P. Wachinou, Nicholas Ngomi

**Affiliations:** ^a^ Department of Epidemiology and Public Health, University of Maryland School of Medicine, Baltimore, MD, USA; ^b^ Center for Bioethics and Research, Ibadan, Nigeria; ^c^ Department of Medicine, University of Cape Town, Cape Town, South Africa; ^d^ Sydney Brenner Institute of Molecular Bioscience, University of Witwatersrand, Johannesburg, South Africa; ^e^ Faculté de Médecine et d’Odonstomatologie, Université des Sciences, des Techniques, et des Technologies de Bamako, Bamako, Mali; ^f^ Department of Integrative Biomedical Sciences, Institute of Infectious Disease and Molecular Medicine, University of Cape Town, Cape Town, South Africa; ^g^ Office of Strategic Information and Research, Institute of Human Virology Nigeria, Abuja, Nigeria; ^h^ Department of Primary Care and Public Health, University of Cambridge, Cambridge, UK; ^i^ Cardiovascular Genetics, Hatter Institute for Cardiovascular Diseases Research in Africa, Department of Medicine, University of Cape Town, Cape Town, South Africa; ^j^ National Livestock Resources Research Institute, Tororo, Uganda; ^k^ Clinical Lab Molecular Virology Unit, Institute of Human Virology Nigeria, Abuja, Nigeria; ^l^ Division of Medical Microbiology, University of Cape Town, Cape Town, South Africa; ^m^ Department of Parasitology, Noguchi Memorial Institute for Medical Research, University of Ghana, Accra, Ghana; ^n^ National Hospital for Tuberculosis and Pulmonary Diseases, Cotonou, Benin Republic; ^o^ Health Challenges and Systems program, African Population and Health Research Center, Nairobi, Kenya

**Keywords:** Africa, genomics, H3Africa, research implementation, challenges

## Abstract

**Background**: There is exponential growth in the interest and implementation of genomics research in Africa. This growth has been facilitated by the Human Hereditary and Health in Africa (H3Africa) initiative, which aims to promote a contemporary research approach to the study of genomics and environmental determinants of common diseases in African populations.

**Objective**: The purpose of this article is to describe important challenges affecting genomics research implementation in Africa.

**Methods**: The observations, challenges and recommendations presented in this article were obtained through discussions by African scientists at teleconferences and face-to-face meetings, seminars at consortium conferences and in-depth individual discussions.

**Results**: Challenges affecting genomics research implementation in Africa, which are related to limited resources include ill-equipped facilities, poor accessibility to research centers, lack of expertise and an enabling environment for research activities in local hospitals. Challenges related to the research study include delayed funding, extensive procedures and interventions requiring multiple visits, delays setting up research teams and insufficient staff training, language barriers and an underappreciation of cultural norms. While many African countries are struggling to initiate genomics projects, others have set up genomics research facilities that meet international standards.

**Conclusions**: The lessons learned in implementing successful genomics projects in Africa are recommended as strategies to overcome these challenges. These recommendations may guide the development and application of new research programs in low-resource settings.

## Background

African populations are characterized by greater levels of genetic diversity, extensive population substructure, and less linkage disequilibrium among loci compared to non-African populations [1–8]. Nonetheless, most of our knowledge of the human genome is based on studies from populations of European ancestry, due to lack of investment in exploring genomics in Africa. Understanding the levels and patterns of variation in African genomes, together with phenotype data on variable traits, including susceptibility to disease, is critical for reconstructing modern human origins, the genetic basis of adaptation to diverse environments, and the development of effective therapeutic agents for diseases [8].

In 2010, the National Institutes of Health and the Wellcome Trust established the Human Hereditary and Health in Africa (H3Africa) Initiative, which aims to facilitate a contemporary research approach to the study of genomics and environmental determinants of common diseases, with the goal of improving the health of African populations [9]. However, the conduct of these genomics research projects has been fraught with challenges that affect participation and outcome. These challenges are encountered throughout a project’s life cycle, but especially in the planning and implementation phases. Some of the problems encountered and strategies to overcome them are discussed here.

## Resources

A major impediment to the implementation of genomics research in Africa is the dearth of resources in general. These include lack of expertise in specific fields, poorly equipped facilities, inadequate infrastructure and erratic power supply. At the planning phase, site visits should be conducted to identify potential drawbacks. Such visits should include feasibility assessments to detect the limitations of potential study sites, especially basic physical and organizational structure required to implement the research. The outcome of the site visits should guide final site selection and budget development. Where expertise in a unique field is not available at a local institution, effort should be made to extend collaborations through calls at society meetings, conferences, personal and institutional networks, and collaborations with large consortia.

With support from H3Africa, infrastructures for genomics research have been set-up in several locations across Africa [10]. With proper maintenance and constant funding, these infrastructures could become well established and used to generate results that will contribute to the knowledge gap of genomics in African populations. However, the burden of maintaining the newly established infrastructure lies with the research stakeholders, particularly the African scientists. Conducting innovative projects, with high rigor and reproducibility would bolster future applications for funding from international agencies. Partnering with local governments and the private sector to invest in research and build upon the current foundation may encourage highly trained scientists to continue conducting competitive research in Africa, train young scientists who become independent investigators and repeat the process. Thus, reduce the ongoing brain drain of scientists from Africa and build sustainable research capacity on the continent.

## Delayed funding

Typically, research funds are released from the sponsor to the research institution on or around the target study start date. In African studies receiving funds from international sponsors, country specific legal and regulatory control policy restraints often lead to delayed transfer of funds to the research institution. Ultimately, research contracts with staff and local facilities are delayed, site operations, recruitment targets, study timelines are negatively impacted and research staff may move on to other projects.

Even when anticipated, delayed funding may be difficult to overcome due, for example, to new government financial policies. However, it is often helpful if the principal investigator works closely with his/her local institution to develop standard operating procedures that address financial contract agreements and transfer of funds between the sponsor and the recipient’s research organization. Before finalizing financial agreements, the principal investigator or designated financial officer should clarify all relevant funding requirements and restrictions, and inform the sponsor of any potential sources of delay to receiving funds and commencing the study.

## Study coordination

The genomics study coordinator is an important interdisciplinary team member who is multitasked with training and supervising research personnel, overseeing adherence to the study documents and consulting with research stakeholders regularly, to ensure the research objectives are accomplished in a timely manner. In several research projects, study sites are selected, budgets are finalized and protocols are developed before the study coordinator is engaged. In many African countries, the selected sites may not have been visited prior to the planning phase and the study protocol was developed by individuals not involved with the day-to-day management of the study. Thus, minute details may be overlooked and the budget is underestimated. The coordinator is therefore presented with a *fait accompli* while striving to implement the project according to the protocol, with limited funds. Hence, it is imperative to involve a study coordinator as early as possible in a research project, to help build the research team, evaluate the project needs and develop the study documents. Recycling staff between projects in an organization rather than employing new individuals makes it easier to have familiar, well-trained and trusted individuals who are accustomed to the inner workings of the organization, the resources and external collaborators related to the research project.

## Training

Poorly trained research team members are detrimental to a research project. The problems with working with insufficiently trained staff are extensive, ranging from recruiting ineligible study participants to incorrect data analysis and interpretation. Before study implementation, field staff should be trained to understand the study protocols, questionnaires and standard operating procedures. Training curriculum should utilize training manuals designed to evaluate pre- and post-training knowledge, have ample duration to encourage discussion between the participants and feedback from the trainees, and not be excessively crowded. Such manuals should be updated as the study evolves. Performance checks should be conducted regularly, to ascertain staff and site are implementing the study according to the protocol. In institutions where the staff turnover rate is high or the research team members are recycled to other projects, training should be conducted periodically, not only at the beginning of the research project. Such trainings will serve as a refresher for older staff and provide needed guidance for new ones. Small pilot studies, where feasible, may also be useful to practice using the instruments developed before rolling out the main study.

To build research capacity, African scientists can be trained in partnership with mentors in high-income countries. Given that some research team members take on more than one role, for example, a staff who recruits study participants may also be required to enter data into a database, staff may benefit from additional training not primarily related to the research protocol. Such training could include time management, task delegation, communication and networking. Team science, which entails team members with training and expertise in different health profession fields working together to combine and integrate their knowledge, skills, and perspectives into single research projects that are clinically focused [11–13], has been shown to be an impactful and collaborative research practice [14] which would be beneficial in implementing genomics studies.

## Ethics

Recruitment for genomic studies presents considerable ethical challenges in most African countries. In general, members of Institutional Review Boards (IRB) across Africa have reviewed a relatively small number of protocols for genomics research. Their inexperience with such studies and the novelty of the proposed procedures may lead to delays with issuing approval. Many African investigators underestimate the duration of IRB reviews and may be oblivious of the rules governing ethical bodies related to their research sites, such as whether the application for review should be submitted to a national or local IRB and the documents to be included in the application packet. These issues contribute to the submission of deficient or discrepant documents. Additionally, research team members may not complete required research ethics training. All of these factors lead to delay in IRB approval and the implementation of the research study, particularly in multinational, studies with different review procedures and requirements imposed by ethics committees. In the H3Africa consortium, appropriate research ethics approval for all research projects were obtained based on institutional and national ethical IRB framework, as well as genomic and biorepository regulations across Africa.

Obtaining informed consent for genomics research is a complex and challenging process, especially in populations with ethnolinguistic barriers and low literacy levels. Research staff often struggle with explaining genomic terms in local languages, as these terms are often non-existent, and require explanations using familiar terms that do not always satisfactorily convey the information that researchers need to pass along. Also, in many African communities, family and community leaders have an important influence over the decisions members make. Therefore, the voluntariness and individualization of research participation may need more attention and discernment from researchers. Furthermore, there appears to be a widespread misconception among participants, that genetic research studies will provide immediate results. While this may be an understandable position, most studies are unable to provide immediate, clinically relevant and actionable results to participants. This challenge is further heightened by the absence of genetic counselors in these low-resource settings, which could lead to misinterpretation of results causing psychological harm.

To overcome ethical challenges in genomics research, we recommend that informed consent materials are translated to local languages and back translated, to ensure they do not lose their original meaning. Cartoons and videos in local languages may be used to demonstrate the study procedures. Research teams should include a member with adequate knowledge of ethical guidelines that are relevant to the environment where the project will be implemented. All members of the research team should undertake the required ethics training and ethical procedures should be factored into the study timeline and research proposal. Governance frameworks that establish compliance with local to international health regulations, protect intellectual property and data ownership, should be developed and incorporated. Engagement programs, which include local community stakeholders should be employed. Several projects in the H3Africa consortium have implemented these strategies with excellent results.

## Laboratory

Many African institutions lack adequate laboratory facilities and well-trained personnel [15–17] to implement genomics research. Most of the laboratory materials required for genomics research in Africa have to be imported. Because of lengthy procurement procedures and the high cost of materials due to shipping charges and profit margins added by suppliers, laboratory supplies to African countries are often delivered late. Some helpful strategies to ameliorate these problems include engaging African governments to invest in genomics research by providing resources to improve local infrastructure. Governments can also help by relaxing the procurement procedures on laboratory materials and providing adequate remuneration to African scientists, to prevent brain drain.

## Recruitment

Enrolling an adequate number of eligible candidates in genomics research is essential to obtaining sufficient power for reliable results [18]. However, recruitment is often slower or more difficult than originally envisaged. Investigators must always take into account over estimation of potential participants from collaborating sites when deciding on the number of centers and sample size per site [19]. In a review of randomized controlled trials, only 31% of trials achieved their original recruitment target and 50% required an extension to achieve adequate sample size [20]. There may be several reasons for these delays. First, poor infrastructure, due to lack of enabling environment for research activities in local hospitals and accessibility to research centers. Second, the nature of the research, which may involve extensive procedures and interventions requiring multiple visits. Third, barriers to effective communication and limited experience of the researchers. Fourth, participants’ mistrust of the research team and fear of adverse events [21] may also play a role. Other challenges encountered during recruiting participants include the potential for psychological harm that may result from the collection of information on painful experiences or concern about research results. Social harm such as inadvertent disclosure of health status based on study inclusion and exclusion criteria such as HIV status may also occur. Participants may also suffer economic loss for the time spent away from gainful employment while participating in research study procedures. Extensive study procedures can lead to participant fatigue and loss to follow-up, particularly in studies with multiple visits. Language barriers may hinder completion of self-administered questionnaires.

Several strategies may help mitigate these challenges. Optimizing the approach to reach the target population increases the study participation rate. Media that can help create awareness of the study include newsletters, posters, information leaflets in local clinics and hospitals, cartoon illustration of recruitment materials and study procedures. Social messaging and chat fora, radio interviews, presentations at meetings and conferences have been shown to be useful in the H3Africa consortium. For the participants, incentives that are not coercive, may be used to affect participation and retention rates positively. For example, compensating participants for the cost of transportation to the study site or providing vehicles to transport them from communities to study centers. Participation may also be promoted by staggering the study procedures over several visits, so participants are not overwhelmed at each visit. Developing questionnaires in languages relevant to the study population, using investigator-administered questionnaires and collecting data electronically using tablets, with skip patterns would save time. Providing relevant research findings to the participants in a timely manner would also foster trust and aid participant retention. For the researchers, adequate training and motivation to boost their confidence and equip them with the skills needed to manage participants Rewarding field staff with appropriate prizes, for example, travel awards to meetings is also helpful. Frequent communication within the study team and between the study team, and the stakeholders should be established.

## Community engagement

Recruitment of study participants depends to a large extent on buy-in from local communities in the target population [22]. Therefore engaging community members and stakeholders in the planning phase has become an important aspect of research projects [23]. Collaboration with communities provides a wealth of information that can be incorporated into the study design and implementation, and unique perspectives that provide insight into potential harm and benefits of the research project to the target population. Strategies to engage the community include public town hall meetings, private meetings with community representatives such as chiefs and use of the community advisory boards. Each community engagement process should have safeguards to ensure risk-benefit balance, voluntary individualized participation, equity in health and social variants of health. There is also value in research projects investing in public education activities to improve literacy levels and awareness around research of the general public or for communities they are likely to recruit from in the future. Such activities would make community engagement efforts for specific research studies easier. Last, collaboration with traditional healers, especially for studies involving participants with a high likelihood to consult these healers, may improve recruitment.

## Data sharing

Intellectual property ownership, data and samples utilization are usually not discussed by researchers at the planning phase. While data and sample sharing is essential to accelerating scientific discovery, it presents a number of challenges, one of which is balancing the interests of science, the scientist, and the donor [24]. For example, biobanking and biospecimen research is being integrated into studies in Africa, and samples are available many years after the initial research may have ended. In order to use the samples to explore new hypotheses, the burden of re-consenting donors may delay scientific discovery. Because of the history of slavery, biobanking African samples out of Africa have been a sensitive issue at the participant as well as state level. Nonetheless, a recent qualitative study in Nigeria showed that when participants understand the purpose of biobanking, they believe it is beneficial and are willing to share their samples with other researchers. Also, when participants were asked to choose between broad, tiered, or restricted consent, half of the respondents chose broad consent, while others chose restricted and tiered consent for biobanking. The main reason for choosing tiered consent was a desire to maintain control over the types of research conducted with donated samples [24]. The study highlighted the need to carefully document population attitudes to elements of modern scientific research and the consenting process. The H3Africa Consortium, through its ethical, legal and society issues research projects, including the Indigenous Linguistic and Cultural Concepts of Heritability and Comprehension of Genomics Research in Africa (INDIGENE) Study, has provided a platform to discuss and debate ethical issues that are pertinent to Africans. The outcome of the INDIGENE study will guide the development of policies that will be important in a much wider context for genomic research in developing countries.

## Mhealth

mHealth technologies may be utilized at several phases of research projects, including community engagement, recruitment, monitoring of adherence to study protocols, data collection, study close out and translation of research into practice [25,26]. Studies have reported that use of mHealth as a research tool decreases the potential for recall bias in retrospectively collected data at study visits [27–29]; is a cost-effective method to increase adherence to study protocol instructions when compared to one time in person verbal and written instructions to participants [30]; and is useful in contacting difficult to reach study participants [31].

Several challenges need to be carefully considered prior to implementation of mHealth technologies in genomics research studies. As a tool for translating research into practice, mHealth methods may require collaborations with private telecommunications companies whose interests may not be aligned with the researchers’. There are also challenges of privacy and confidentiality, especially in situations where sensitive personal information is shared by mobile phones, which are owned and used by multiple persons. Although the use of mHealth in genomics research presents many exciting opportunities, careful consideration is required in the design and implementation of this technology to ensure that the lessons learnt from more traditional approaches to the conduct of research are incorporated. Results of focus group discussions conducted with members of the target population, before the main study is implemented, may help guide the suitability of mHealth technologies in genomics research.

## Conclusion

In conducting research projects in Africa, investigators should recognize the potential challenges and multi-dimensional opportunities for landscape change in genetics and genomics epidemiology in Africa. Advancing genomics health education, engaging African governments, communities and private sectors to develop sustainable partnerships would help build research capacity on the continent. Shared leadership, cultural awareness, collaboration and establishing trustworthy relationships among African scientists are some strategies that have been successfully employed in H3Africa, to facilitate implementation of genomics research in African populations.

## Paper context

There is exponential growth in the interest and application of genomics research in Africa. However, the implementation of genomics studies on the continent is fraught with multifarious challenges. This article describes such challenges, including language barriers, ill-equipped facilities, lack of enabling environment and poor accessibility to research centers, and discusses recommendations to overcome these challenges in Africa and other low-resource settings.
